# Salicylic acid signaling controls the colonization behavior of *Colletotrichum tofieldiae* in *Arabidopsis thaliana*

**DOI:** 10.3389/fpls.2026.1770854

**Published:** 2026-04-15

**Authors:** Norhafizah Binti Sidek, Shiho Itoh, Toru Aramaki, Yuji Yamasaki, Hisashi Tsujimoto, Kazumasa Shirai, Kousuke Hanada

**Affiliations:** 1 Department of Bioscience and Bioinformatics, Kyushu Institute of Technology, Iizuka, Fukuoka, Japan; 2 Arid Land Research Center, Tottori University, Tottori, Japan

**Keywords:** *Arabidopsis thaliana*, *Colletotrichum tofieldiae*, phosphate availability, salicylic acid signaling, symbiosis

## Abstract

Plant–microbe interactions strongly influence plant growth and nutrient acquisition, and their outcomes depend on nutrient availability. The root endophyte *Colletotrichum tofieldiae* (*Ct*) promotes growth in *Arabidopsis thaliana* under inorganic phosphate (Pi) limitation, but its effects under Pi sufficiency and the role of salicylic acid (SA) signaling remain unclear. Here, we examined Pi-dependent growth responses, nutrient accumulation, and SA signaling in wild-type (WT) and SA-deficient *ics1* mutant plants co-cultivated with *Ct* under low, moderate, and high Pi conditions (25, 150, and 625 µM). Under low Pi, *Ct* significantly enhanced WT growth, increasing leaf number and root length by 41.8% and 50.5%, respectively, and promoting biomass accumulation, with fresh and dry weight increases of 104% and 232% relative to uninoculated controls. Growth promotion was reduced at moderate Pi and shifted toward growth suppression under high Pi. Elemental profiling using inductively coupled plasma mass spectrometry (ICP-MS) revealed pronounced *Ct*-mediated nutrient accumulation under Pi limitation. At low Pi, phosphorus content increased by 281%, accompanied by significant increases in K (70.1%), S (84.5%), and Ca (73.2%). In contrast, at moderate and high Pi, *Ct* consistently enhanced P accumulation, while changes in K, S, and Ca were not significant. *Ct* colonization induced expression of the SA-responsive marker gene *PR1*, particularly under low Pi. In contrast, *ics1* mutants failed to exhibit *Ct-*induced growth promotion and instead displayed growth suppression across all Pi conditions. Together, these findings demonstrate that Pi availability and ICS1-mediated SA biosynthesis jointly determine the outcome of the *Arabidopsis*–*Ct* interaction.

## 1 Introduction

Plants inhabit complex soil ecosystems populated by diverse microorganisms that can either promote or inhibit their growth depending on the environmental conditions (Hassani et al., 2018; Plett and Martin, 2018; Zobel et al., 2024). Under nutrient-limited conditions, numerous plants rely on microbial partners to enhance nutrient availability and enable growth in low-fertility soils (Chhabra and Dowling, 2017). Among these nutrients, inorganic phosphate (Pi) plays a particularly critical role in shaping plant–microbe interactions (Castrillo et al., 2017; Inoue et al., 2024). Because Pi is largely insoluble and poorly mobile in the soil, plants under low-Pi conditions frequently depend on arbuscular mycorrhizal fungi or root endophytes to mobilize and transport Pi. During this mutualistic exchange, fungi retain part of the absorbed Pi while delivering another portion to their host in return for photosynthetically fixed carbon (Chiu and Paszkowski, 2019; Mehta et al., 2019).

Unlike most terrestrial plants, members of Brassicaceae, including *Arabidopsis thaliana*, have lost their ability to form classical mycorrhizal associations (Cosme et al., 2018; Delaux et al., 2014; Sharma et al., 2023). However, *Arabidopsis* can establish a mutualistic relationship with the root endophyte *Colletotrichum tofieldiae* (*Ct*), which was originally identified in natural populations inhabiting Pi-deficient soils in central Spain (Hiruma et al., 2016). Although several *Colletotrichum* species are pathogenic (Baroncelli et al., 2016; O'Connell et al., 2012), *Ct* can promote host growth and Pi uptake under nutrient limitation. Hiruma et al. (2016) demonstrated that Pi availability determines the outcome of this interaction; under low-Pi conditions, *Ct* acts as a beneficial symbiont, whereas under high-Pi conditions, the relationship may shift away from mutualism. They further showed that the plant phosphate starvation response (PSR) and PEN2-dependent indole glucosinolate–linked immunity collaboratively regulate this balance. However, it remains unclear whether *Ct* retains its beneficial role under Pi-sufficient conditions. Additionally, the contribution of the central immune pathway governed by salicylic acid (SA) signaling to this interaction has not been elucidated.

SA signaling is best known for activating defense responses and establishing systemically acquired resistance against pathogens (Kumar, 2014; Kunkel and Brooks, 2002). Within this pathway, *Pathogenesis-Related 1* (*PR1*) serves as a robust transcriptional marker of SA-mediated immune activation (Jain and Khurana, 2018; Javed et al., 2025). SA biosynthesis depends primarily on Isochorismate Synthase 1 (ICS1), and the disruption of this enzyme leads to reduced SA accumulation and compromised immune signaling (Garcion et al., 2008; Wildermuth et al., 2001). Although the role of SA in plant–pathogen interactions is well defined, emerging studies indicate that SA signaling also affects plant associations with endophytic fungi (Bastias et al., 2018; Benjamin et al., 2022; Kou et al., 2021). However, the mechanisms through which SA influences plant interactions with endophytic fungi remain unclear. Therefore, understanding whether ICS1-dependent SA biosynthesis and PR1-mediated signaling contribute to maintaining a stable interaction with *Ct* remains an important unanswered question.

In this study, we investigated how Pi availability and SA signaling jointly influenced the outcome of the *Arabidopsis*–*Ct* association. Using wild-type (WT) plants and an SA-deficient *ics1* mutant, we assessed fungal colonization, nutrient accumulation, and host immune responses under various Pi conditions. Taken together, our findings revealed that SA signaling functions as a regulatory mechanism that restricts fungal proliferation and maintains compatibility under low Pi, thereby preventing the mutualistic relationship from shifting toward pathology.

## 2 Materials and methods

### 2.1 Plant materials and growth conditions

Seeds of *A. thaliana* wild-type (WT, Col-0) and SA-deficient *ics1* knockout mutants were surface-sterilized with hypochlorous acid and vernalized at 4 °C for 2 days. Seedlings were grown on ½-strength Murashige and Skoog (MS) medium prepared from individually measured analytical-grade reagents (FUJIFILM Wako Pure Chemical Corporation, Osaka, Japan; Dojindo Laboratories, Kumamoto, Japan), according to the composition listed in **Supplementary Table S1**. The medium was supplemented with 1% (w/v) sucrose as the carbon source, 0.05% (w/v) MES buffer (pH 5.7), B5 vitamins, and 0.8% (w/v) agar before autoclaving. Plants were cultivated in a controlled-environment chamber (BioTRON, NK System, Japan) at 22 °C under long-day conditions (16 h light/8 h dark) with a light intensity of 76 µmol m^−2^ s^−1^ provided by three fluorescent lamps (Mitsubishi Electric, Japan).

### 2.2 Fungal culture and spore preparation

The endophytic fungus *Ct* (Hiruma et al., 2016) was cultured in a darkened climate chamber at 25 °C, 50% relative humidity, and under constant black-light irradiation to promote sporulation. Mathur’s medium was used for fungal growth, as described by Hiruma et al. (2016). After seven days of incubation, fungal colonies were collected into sterile 2 mL microcentrifuge tubes using a sterile 1 mL pipette tip. Spores were detached by vortexing and filtered through a 40 µm cell strainer (Falcon^®^, Corning) to remove hyphal fragments, yielding a clean spore suspension. Spore concentration was determined using a Thoma hemocytometer (Sunlead Glass Corp., Saitama, Japan), and the suspension was adjusted with sterile Milli-Q water to a final concentration of 1 × 10^6^ spores per 100 mL for subsequent inoculation.

### 2.3 Establishment of phosphate-defined infection media

For infection assays, ½-strength MS medium was prepared as described above and adjusted to three Pi concentrations by modifying the KH_2_PO_4_ content to 0.34, 2.04, and 8.50 g L^−1^, corresponding to 25, 150, and 625 µM Pi, respectively. These concentrations were chosen to represent low, moderate, and high Pi availabilities, respectively. Media were autoclaved at 121 °C for 20 min and cooled below 50 °C before inoculation. *Ct* inoculation was performed by homogeneously mixing the standardized spore suspension into the molten medium before plate solidification. Control plates received an equal volume of sterile Milli-Q water.

Five-day-old *Arabidopsis* WT and *ics1* knockout seedlings were transferred onto phosphate-defined media and co-cultivated with *Ct* under long-day conditions for three weeks. Each treatment comprised six independent biological replicates (n = 6 seedlings per treatment), with each seedling originating from a separate plate. Individual seedlings were used directly for growth measurements.

Plant growth was assessed by measuring fresh weight, dry weight (after drying at 60 °C for 24 h), leaf number, and root length using ImageJ software (NIH, USA). For molecular analyses, plants were harvested after 10 days to capture early transcriptional and symbiotic responses, whereas elemental measurements were performed on 3-week-old plants.

### 2.4 Elemental analysis by inductively coupled plasma tandem mass spectrometry

Harvested tissues were stored at −80 °C and subsequently sent to the Molecular Breeding Laboratory, Arid Land Research Center, Tottori University (Japan) for elemental analysis. The samples were freeze-dried for 24 h using a TAITEC VD-550R freeze dryer (Taitec Corporation, Japan), transferred to quartz inserts, and weighed to determine the dry biomass. Each sample was digested in 5 mL concentrated nitric acid (Wako, Japan) using a microwave digestion system (ETHOS UP, Milestone General, Italy). Next, over the course of 20 min, the digestion program reached 190 °C and was maintained at this temperature for 10 min. Following digestion, the volume of the solution was increased to 5 mL by the addition of nitric acid. The samples were diluted 1:4000 with ultrapure water and analyzed in duplicate using an inductively coupled plasma mass spectrometry instrument (Agilent 8900, Agilent Technologies, USA). The concentrations of phosphorus (P), potassium (K), sulfur (S), and calcium (Ca) were quantified using multi-element calibration standards. Data quality was ensured by excluding measurements with a relative standard deviation (RSD) > 25%. Elemental analysis was performed using three independent biological replicates per treatment.

### 2.5 Quantitative reverse transcription PCR analysis

Total RNA was isolated from *Arabidopsis* WT seedlings co-cultivated with *Ct* using Plant RNA Reagent (Thermo Fisher Scientific, USA). Complementary DNA (cDNA) was synthesized using the QuantiTect^®^ Reverse Transcription Kit (QIAGEN, Germany) following the manufacturer’s protocol. qRT-PCR was carried out using SsoFast™ EvaGreen^®^ Supermix (Bio-Rad, USA) on an Mx3005P System (Agilent Technologies, USA). Expression of the SA-responsive gene *PR1* was quantified, and transcript levels were normalized to the *Arabidopsis* reference gene *AtACT2*. Fungal biomass in *Arabidopsis* roots was quantified by measuring the ratio of *CtTUB* (fungal β-tubulin) to *AtACT2*, which reflects the relative abundance of fungal tissue relative to plant tissue. Relative transcript levels for all assays were calculated using the ΔCt method (Livak and Schmittgen, 2001). qRT-PCR analyses were performed using four independent biological replicates. The primer sequences are listed in **Supplementary Table S2**.

## 3 Results

### 3.1 Pi-dependent effects of *Colletotrichum tofieldiae* on *Arabidopsis* growth

**Figure 1:**
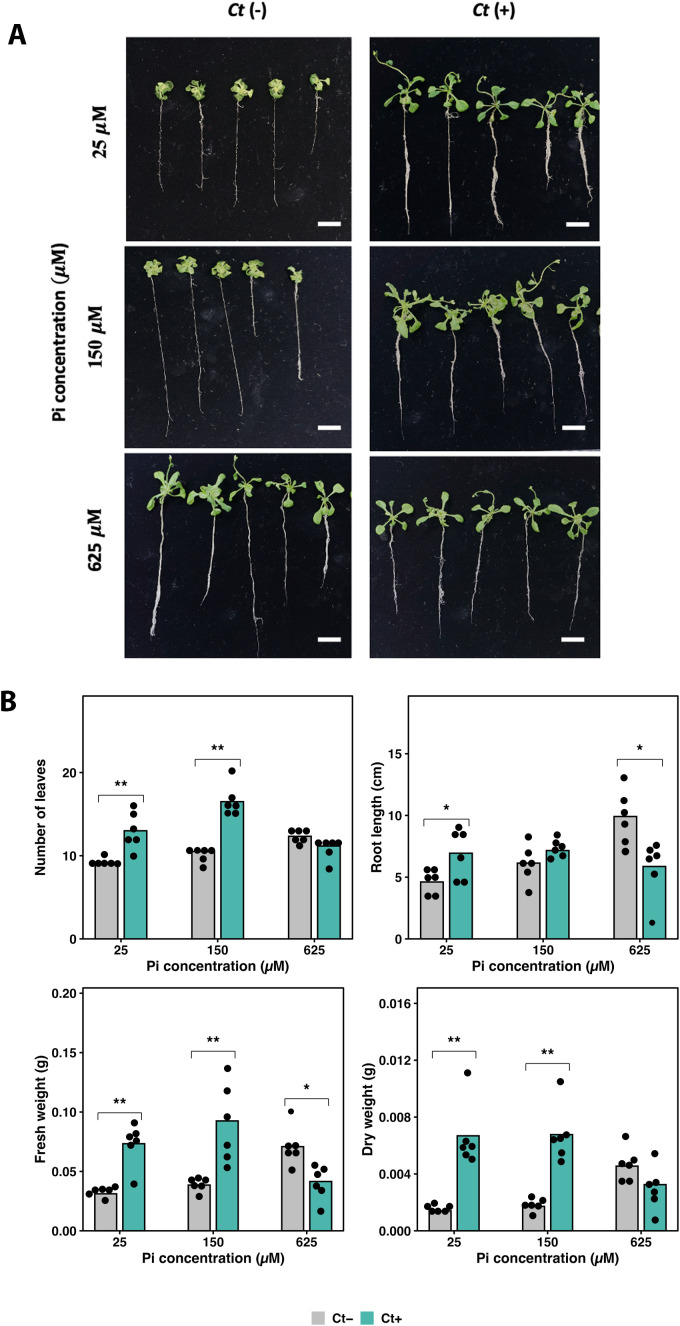
Pi-dependent effects of *Colletotrichum tofieldiae* (*Ct)* on *Arabidopsis* growth. **(A)** Representative images of Wild-type (WT) plants grown with (*Ct* +) or without (*Ct* −) fungal inoculation under Pi concentrations of 25, 150, and 625 µM for 3 weeks. Scale bars = 1 cm. **(B)** Quantitative growth parameters showing number of leaves, root length, fresh weight, and dry weight of *Ct* + and *Ct* − plants across Pi treatments. Bars represent mean values (n = 6). Black dots indicate individual replicates. Asterisks denote significant differences between *Ct* + and *Ct* − within each Pi condition (*p* < 0.05 for *, *p* < 0.01 for **; t-test).

For infection assays, three Pi concentrations were used to represent low, moderate, and high Pi availability (25, 150, and 625 µM, respectively). After three weeks of co-cultivation, *Ct* inoculation significantly enhanced plant growth under low Pi conditions (**Figure 1B**).

At 25 µM Pi, *Ct*-inoculated plants exhibited significant increases of 41.8% in leaf number, 50.5% in root length, 104% in fresh weight, and 232% in dry weight relative to uninoculated controls. At 150 µM Pi, *Ct* inoculation resulted in significant increases in leaf number (57.1%), fresh weight (61.5%), and dry weight (121%), whereas the increase in root length (16.6%) was not statistically significant.

In contrast, under high Pi conditions, *Ct* inoculation was associated with growth suppression. Significant reductions were observed for root length (−40.8%) and fresh weight (−41.4%), while decreases in leaf number (−9.46%) and dry weight (−28.6%) were not statistically significant relative to uninoculated plants (**Figure 1B**). Growth responses are expressed as percentage changes relative to the corresponding controls, consistent with reporting conventions used in previous studies (Boamah et al., 2021).

### 3.2 Nutrient accumulation under *Ct* colonization

**Figure 2:**
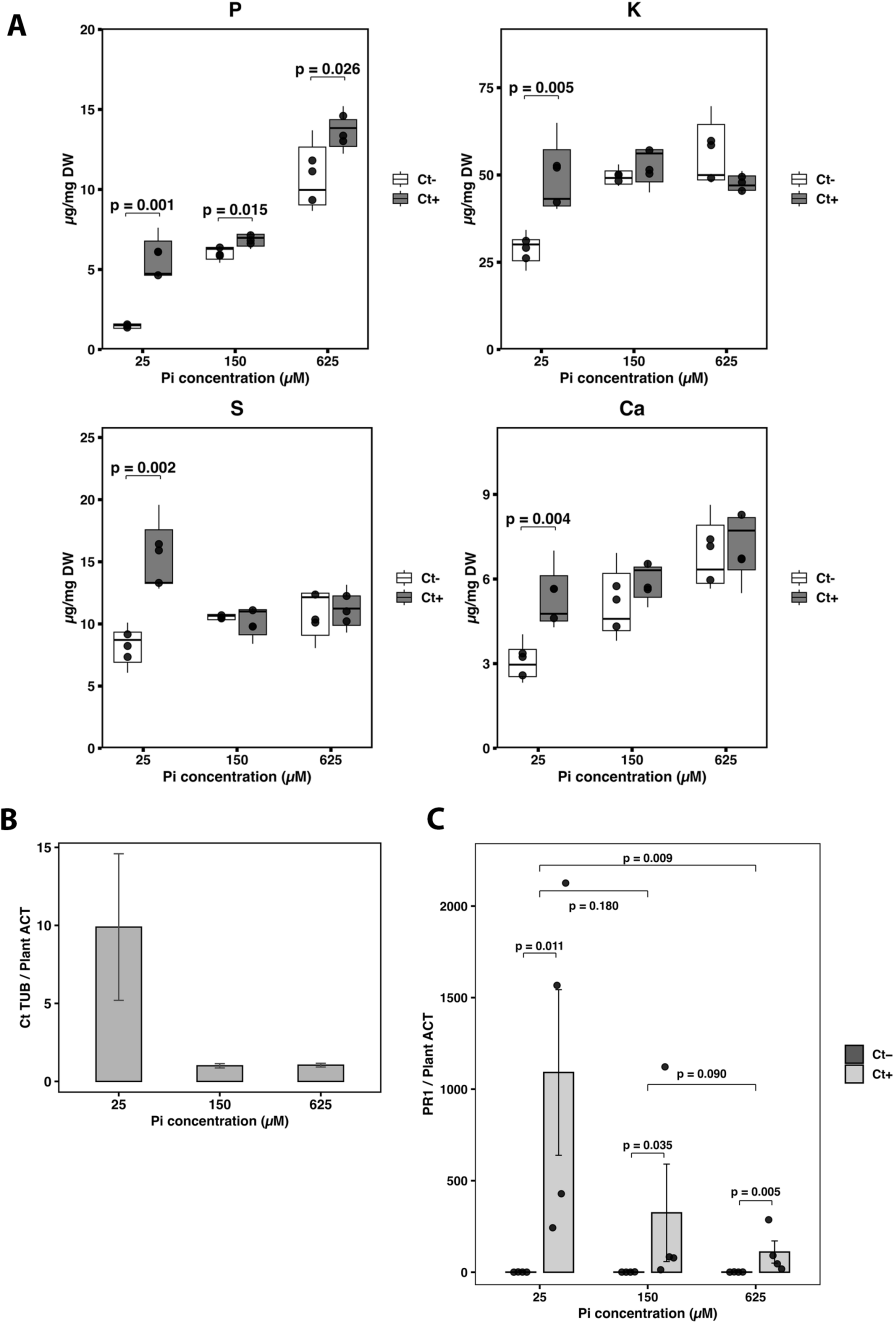
Phosphate-dependent regulation of nutrient accumulation, fungal colonization, and salicylic acid signaling during *Arabidopsis*–Ct interaction. **(A)** Concentrations of phosphorus (P), potassium (K), sulphur (S), and calcium (Ca) in *Arabidopsis* shoots after three weeks of co-cultivation with *Colletotrichum tofieldiae* (*Ct*) under different phosphate (Pi) concentrations (25, 150, and 625 µM). *Ct*− indicates uninoculated plants, and *Ct*+ indicates Ct-inoculated plants. Nutrient concentrations are expressed as µg mg^−1^ dry weight (DW). Bars represent mean values (n = 3). Statistical differences between *Ct*− and *Ct*+ plants within each Pi condition were evaluated using Student’s t-test, and significant *p*-values are indicated. **(B)** Relative fungal biomass of *Ct* in *Arabidopsis* roots after 10 days of co-cultivation under the indicated Pi concentrations. Fungal biomass was quantified by qRT-PCR using *CtTUB* as the fungal marker gene and normalized to *Arabidopsis ACT2* (*CtTUB/AtACT2*). Bars represent mean ± SE (n = 4). **(C)** Expression of the salicylic acid–responsive gene *PR1* in Arabidopsis seedlings co-cultivated with *Ct* for 10 days under different Pi concentrations. *PR1* transcript levels were quantified by qRT-PCR and normalized to *AtACT2*. Bars represent mean ± SE (n = 4). Statistical significance between *Ct*- and *Ct*+ plants within each Pi condition was determined using Student’s *t*-test (*p* < 0.05).

Elemental profiling of Arabidopsis shoots after three weeks of co-cultivation with *Ct* was performed using inductively coupled plasma mass spectrometry (ICP-MS) to quantify elemental concentrations (**Figure 2A**). This approach enabled the simultaneous detection of macro- and micronutrients, providing a detailed profile of nutrient accumulation patterns in response to *Ct* colonization. As phosphate deficiency often triggers broader changes in ion homeostasis, additional elements including K, S, and Ca were examined to evaluate whether *Ct*-mediated effects extend beyond phosphorus acquisition.

Under low Pi conditions, Ct inoculation led to a significant increase in multiple elements, including P, K, S and Ca. Phosphorus accumulation increased by 281% relative to uninoculated controls, while K, S, and Ca increased by 70.1%, 84.5%, and 73.2%, respectively. At moderate Pi availability, *Ct* inoculation resulted in a significant increase in P content (13.4%), whereas changes in K, S, and Ca were not statistically significant. Similarly, under high Pi conditions, P content remained significantly higher in *Ct*-inoculated plants (26.9%), while no significant differences were detected for K, S, or Ca. These results demonstrate that Ct consistently enhances phosphorus accumulation across a broad range of Pi conditions, but promotes coordinated multi-element nutrient accumulation predominantly under phosphate-limited conditions.

### 3.3 Fungal colonization under different Pi conditions

To determine whether the extent of *Ct* colonization correlated with the Pi-dependent growth responses observed, the fungal biomass in Arabidopsis roots was quantified using qRT-PCR. The relative fungal abundance was calculated as the ratio of *CtTUB* to *AtACT* transcript levels, providing an estimate of fungal biomass normalized to plant biomass.

Fungal colonization followed a clear Pi-dependent trend across the three Pi concentrations tested. *Ct* biomass was highest under low Pi (25 µM), intermediate at 150 µM Pi, and markedly reduced under high Pi (625 µM), indicating that fungal proliferation is favored under nutrient-limited conditions (**Figure 2B**).

### 3.4 Salicylic acid signaling mediates *Arabidopsis*–*Ct* interaction

**Figure 3:**
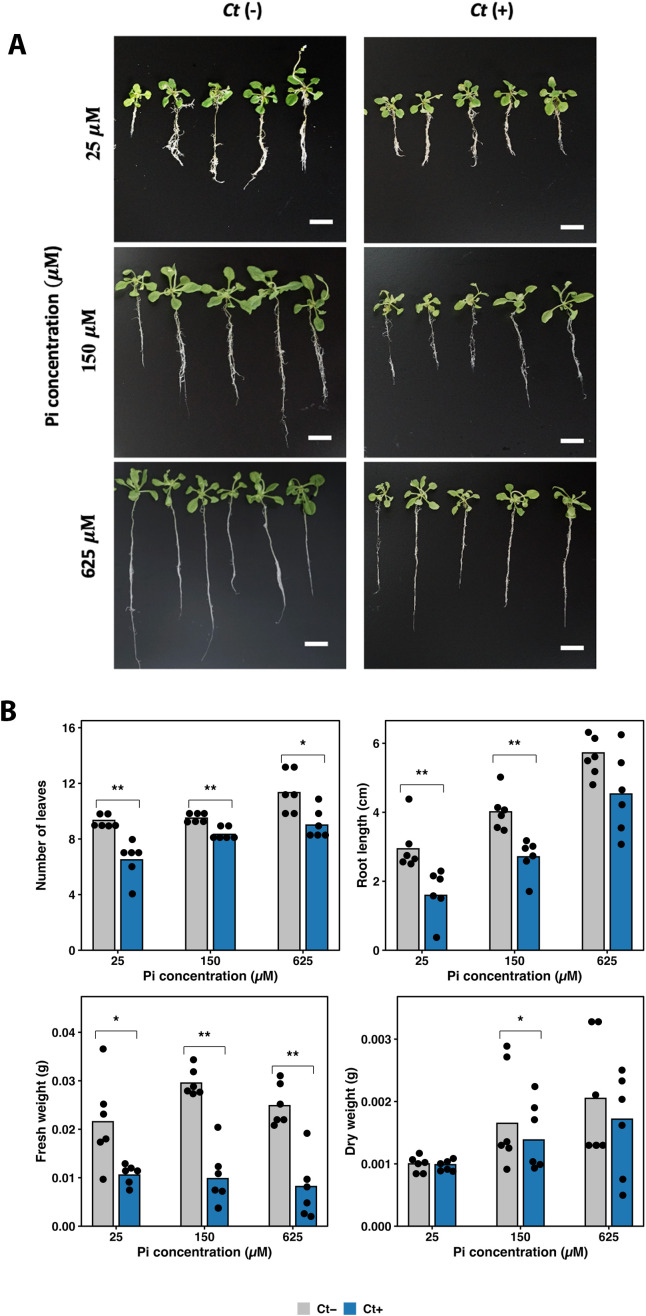
Growth responses of the *ics1* mutant to *Ct* under different Pi levels. **(A)** Representative images of *ics1* plants grown with (*Ct* +) or without (*Ct* −) fungal inoculation under 25, 150, and 625 µM Pi for three weeks. Scale bars = 1 cm. **(B)** Quantitative growth parameters showing leaf number, root length, fresh weight, and dry weight. Bars represent mean values (*n* = 6). Black dots indicate individual seedlings. Asterisks denote significant differences between *Ct* + and *Ct* − within each Pi condition (*p* < 0.05 for *, *p* < 0.01 for **; t-test).

To determine whether SA signaling contributes to the phosphate-dependent *Arabidopsis-Ct* interaction, expression of the SA-responsive gene *PR1* was first analyzed. We hypothesized that changes in SA activity influence the symbiotic responses observed during *Ct* colonization. The results showed that *Ct* colonization strongly induced *PR1* expression under all Pi conditions, with the highest activation observed at low Pi values, indicating that SA was activated during nutrient limitation (**Figure 2C**). To evaluate whether this activation was functionally relevant, we compared SA-deficient *ics1* knockout mutants with and without *Ct* inoculation (**Figure 3**). Prior to this comparison, we directly assessed basal growth differences between uninoculated WT and *ics1* seedlings under the three Pi concentrations tested. Under uninoculated conditions, *ics1* plants exhibited reduced basal growth relative to WT. Root length was significantly shorter in *ics1* at all Pi levels. Leaf number was significantly reduced at 150 µM Pi, while fresh weight was significantly lower at 150 and 625 µM Pi. Dry weight was significantly reduced at 25 and 625 µM Pi (**Supplementary Figure S1**). These results indicate that *ics1* plants display reduced basal growth compared with WT even in the absence of *Ct* inoculation.

*Ct* inoculation resulted in consistent growth suppression in *ics1* plants across all Pi levels (**Figure 3**). Leaf number was significantly reduced at all Pi concentrations, with decreases of 30.4% at 25 µM Pi, 12.3% at 150 µM Pi, and 20.6% at 625 µM Pi. Root length was significantly reduced under low and moderate Pi conditions, declining by 45.9% at 25 µM Pi and 32.5% at 150 µM Pi, whereas the reduction observed at 625 µM Pi (20.8%) was not statistically significant. Fresh weight showed significant reductions across all Pi levels, decreasing by 51.0% at 25 µM Pi, 66.8% at 150 µM Pi, and 67.1% at 625 µM Pi. In contrast, dry weight exhibited a significant reduction only at 150 µM Pi (16.2%), while changes at 25 µM Pi (1.33%) and 625 µM Pi (13.0%) were not statistically significant (**Figure 3B**).

Due to the strongest *Ct* colonization observed under low Pi condition, fungal biomass was specifically measured at 25 µM Pi, as this condition offers the clearest context for evaluating the effects of SA signaling on fungal colonization. **Supplementary Figure S2** shows the relative fungal biomass of *Ct* in *ics1* roots under these low Pi conditions. These data suggest that *Ct* colonization and fungal biomass accumulation were higher in the *ics1* mutant compared to WT plants under low Pi conditions.

Together, these results demonstrate that *ICS1*-mediated SA biosynthesis is required to maintain a beneficial growth response to *Ct* under phosphate-limited conditions. In the absence of functional SA signaling, the *Arabidopsis–Ct* interaction shifts toward a growth-suppressive outcome.

## 4 Discussion

The interaction between *A. thaliana* and *Ct* is strongly dependent on Pi availability (Hacquard et al., 2016; Hiruma et al., 2016). Our results demonstrate that this association is not a simple mutualism, but a dynamic exchange in which both the host and fungus simultaneously benefit and compete for resources. Under low Pi conditions, the *Ct*-colonized plants accumulated higher levels of P, K, S, and Ca, indicating that the host acquired nutrients from the fungus when external Pi was scarce. At the same time, fungal biomass was the greatest under low Pi conditions, suggesting that *Ct* proliferated more efficiently when the host was nutrient-limited and likely supplied more carbon. This is consistent with the carbon exchange mechanisms observed in other beneficial endophytes (Behie et al., 2017; Howard et al., 2024; Hoysted et al., 2023). Under high Pi, this reciprocal exchange collapses and *Ct* biomass declines sharply, likely because the plant withdraws carbon support when additional nutrient acquisition is unnecessary. Thus, even during its growth-promoting phase, the interaction reflects a form of regulated antagonism, in which benefits exist only under favorable environmental conditions that maintain a balance between fungal colonization and host defense (Schulz and Boyle, 2005).

SA-dependent immune signaling is a key regulatory component of this balance. In our study, *PR1* expression, a canonical marker of SA pathway activation, was consistently induced during *Ct* colonization across all Pi conditions. This observation is consistent with previous reports demonstrating that *PR1* is a reliable marker of SA-dependent immune activation in plant–microbe interactions (Loon et al., 2006; Mengistu, 2020; Javed et al., 2025). SA is a central immune hormone required for activating defense responses against microbial invasion (Kunkel and Brooks, 2002; Zhang and Li, 2019). Notably, the highest *PR1* induction occurred under low Pi conditions, the same conditions under which *Ct* strongly promoted growth and proliferated most extensively. This indicates that *Arabidopsis* maintains active SA-mediated immune surveillance even during beneficial interactions, likely restricting excessive fungal proliferation and preventing a transition toward pathogenic behavior. Conversely, weaker *PR1* induction under high Pi conditions reflects reduced interaction intensity when nutrient exchange is unnecessary.

Nutrient availability, particularly phosphate status, is increasingly recognized as a regulator of SA signaling and immune outputs. Castrillo et al. (2017) reported that phosphate starvation reshapes root immune responses through microbiota-dependent mechanisms, linking Pi availability to immune regulation. Similarly, Chan et al. (2021, 2022) showed that external phosphate supply influences SA-dependent defense gene activation. Consistent with these findings, we observed that SA-associated responses varied with Pi availability, with the strongest activation occurring under phosphate limitation. Furthermore, recent work has shown that SA biosynthesis and signaling contribute to maintaining controlled interactions with microbial partners by balancing colonization and defense activation (Sommer et al., 2024). In accordance with this regulatory role of SA, we found that the SA-deficient *ics1* mutant, which lacks isochorismate synthase 1 and is impaired in SA biosynthesis (Wildermuth et al., 2001), failed to sustain growth promotion under low Pi and exhibited increased fungal biomass relative to WT. Similar effects have been reported in *Piriformospora indica–Arabidopsis* interactions, where loss of SA signaling (*sid2/ics1*, *npr1*) results in excessive colonization (Jacobs et al., 2011). These findings indicate that SA biosynthesis is required to maintain compatibility and prevent uncontrolled fungal expansion under nutrient limitation.

Together, our data support a model in which Pi availability defines the physiological context of the *Arabidopsis–Ct* interaction, whereas SA signaling provides a regulatory mechanism that keeps the association beneficial under nutrient limitation. Under low Pi conditions, reciprocal nutrient and carbon exchange lead to mutual benefits, but only because SA restricts fungal proliferation. Under high Pi conditions, nutrient exchange appears to be reduced, and the growth-promoting effect of *Ct* is no longer observed, suggesting a shift toward a more competitive interaction. While previous studies have established the Pi-dependent nature of the *Arabidopsis–Ct* association, our results provide functional evidence that SA signaling contributes to regulating the balance between host growth and fungal proliferation in response to nutrient status. How phosphate availability mechanistically influences SA-dependent immune regulation during endophytic colonization remains an important direction for future research.

## Data Availability

The original contributions presented in the study are included in the article/**Supplementary Material**. Further inquiries can be directed to the corresponding author.
