# Supplementary material for: Salicylic acid signaling controls the colonization behavior of Colletotrichum tofieldiae in Arabidopsis thaliana
Source: Front Plant Sci. 2026 Apr 15;17:1770854. doi: 10.3389/fpls.2026.1770854 (PMC13124722; doi:10.3389/fpls.2026.1770854)
Supplement: Supplementary file 1 [file DataSheet1.pdf]

Supplementary Figure

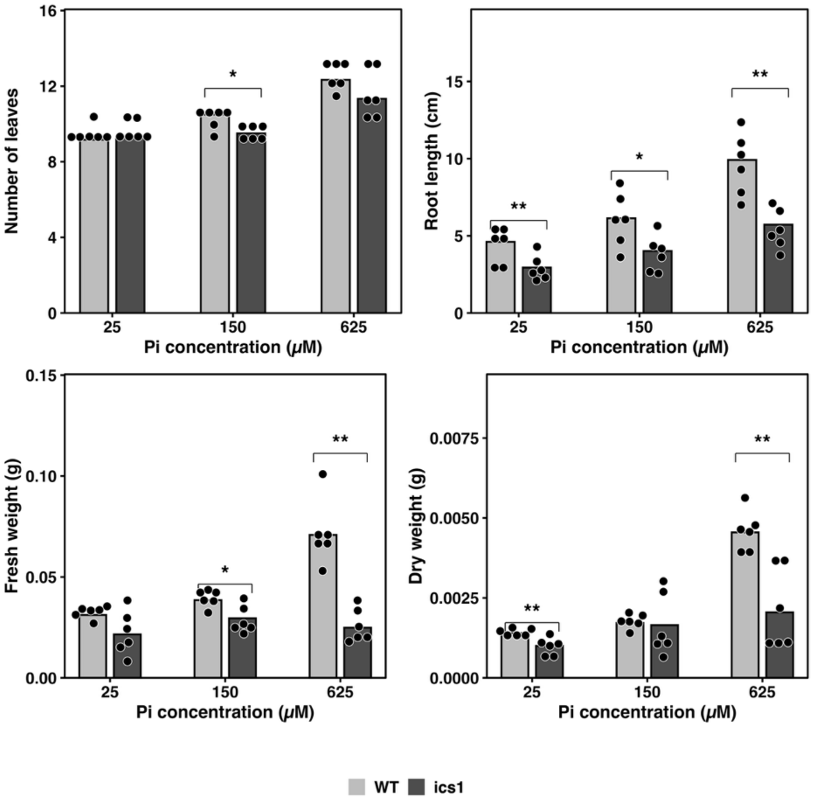

Supplementary Figure S1. Comparison of growth parameters between uninoculated WT and *ics1* seedlings under low (25 µM), moderate (150 µM), and high (625 µM) Pi conditions. Data represent six biological replicates (six seedlings from independent plates). These data show that *ics1* plants display reduced basal growth compared with WT even in the absence of *Ct* inoculation.

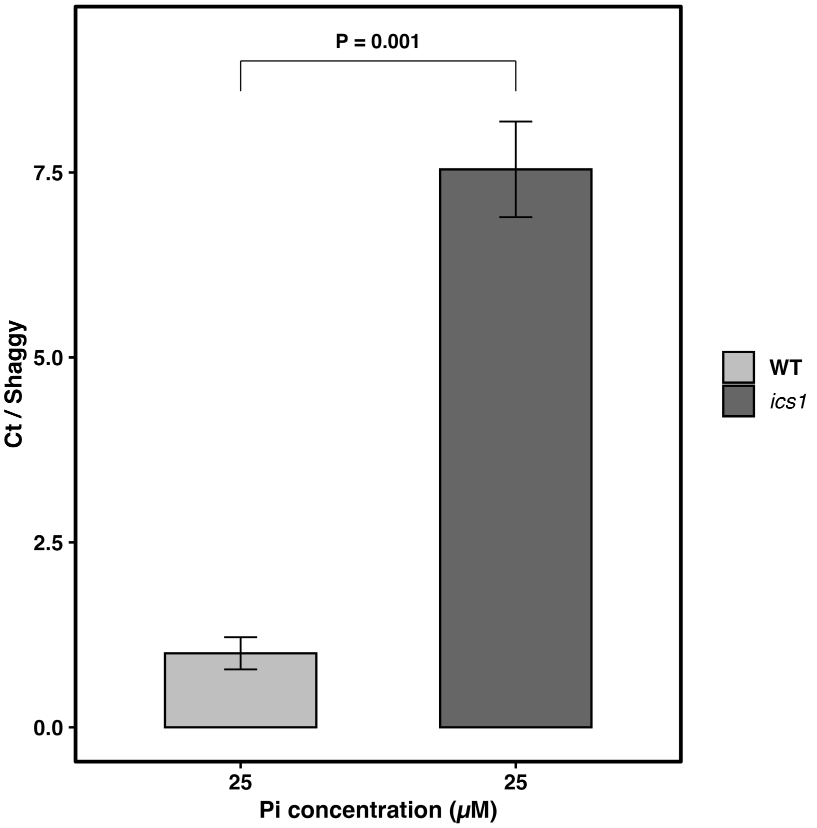

Supplementary Figure S2. Relative fungal biomass of *Ct* in the *ics1* mutant under low phosphate conditions. Relative fungal biomass of *Ct* in *Arabidopsis ics1* roots after 10 days of co-cultivation under low phosphate (25 µM Pi). Fungal biomass was quantified by qPCR using *CtTUB* as the fungal marker gene and normalized to the reference gene (*CtTUB/Shaggy*). Bars represent mean ± SE (n = 4). Statistical significance was assessed using Student's *t*-test.

Supplementary Table S1. Composition of ½ MS medium.

**Reagent**	**Amount (g L^{-1})**
NH_4NO_3	0.825
KNO_3	0.95
KH_2PO_4	0.085
$CaCl_2 \cdot 2H_2O$	0.22
$MgSO_4 \cdot 7H_2O$	0.185
$MnSO_4 \cdot 5H_2O$	0.01115
$ZnSO_4 \cdot 7H_2O$	0.0043
H_3BO_3	0.0031
KI	0.000415
$Na_2MoO_4 \cdot 2H_2O$	0.000125
$CuSO_4 \cdot 5H_2O$	0.0000125
$CoCl_2 \cdot 6H_2O$	0.0000125
$FeSO_4 \cdot 7H_2O$	0.013
Na_2–EDTA	0.01865
Inositol	0.1
Nicotinic acid	0.0002
Glycine	0.002
Pyridoxine HCl	0.0005
Thiamine HCl	0.0001
Agar	8.0

Supplementary Table S2. Primer sequences used for qRT-PCR analysis of host and fungal genes.

**Name**	**Sequence (5′ → 3′)**
PR1_F	CGAGAAGGCTAACTACAATCACG
PR1_R	ACACCTCACTTTGGGCACATC
Plant Actin_F	ACCTTGCTGGACGTGACCTTACTGAT
Plant Actin_R	GTTGTCTGCTGTTTCCAGCGTTT
Ct tubulin beta-1 chain_F	GCGGGAAAGTGTCAATGAAG
Ct tubulin beta-1 chain_R	GACTCTGCGGAAACTGTTCTG
